# Physiological Conditions and dsRNA Application Approaches for Exogenously induced RNA Interference in *Arabidopsis thaliana*

**DOI:** 10.3390/plants10020264

**Published:** 2021-01-30

**Authors:** Konstantin V. Kiselev, Andrey R. Suprun, Olga A. Aleynova, Zlata V. Ogneva, Alexandra S. Dubrovina

**Affiliations:** 1 Laboratory of Biotechnology, Federal Scientific Center of the East Asia Terrestrial Biodiversity, Far Eastern Branch of the Russian Academy of Sciences, 690022 Vladivostok, Russia; kiselev@biosoil.ru (K.V.K.); SUPRUN@biosoil.ru (A.R.S.); aleynova@biosoil.ru (O.A.A.); ogneva@biosoil.ru (Z.V.O.); 2 The School of Natural Sciences, Far Eastern Federal University, 690090 Vladivostok, Russia

**Keywords:** exogenously induced RNA interference (exo-RNAi), external dsRNA, foliar application, plant gene silencing, transgene, *Arabidopsis thaliana*

## Abstract

Recent studies have revealed that foliar application of double-stranded RNAs (dsRNAs) or small-interfering RNAs (siRNAs) encoding specific genes of plant pathogens triggered RNA interference (RNAi)-mediated silencing of the gene targets. However, a limited number of reports documented silencing of plant endogenes or transgenes after direct foliar RNA application. This study analyzed the importance of physiological conditions (plant age, time of day, soil moisture, high salinity, heat, and cold stresses) and different dsRNA application means (brush spreading, spraying, infiltration, inoculation, needle injection, and pipetting) for suppression of neomycin phosphotransferase II (*NPTII*) transgene in *Arabidopsis thaliana*, as transgenes are more prone to silencing. We observed a higher *NPTII* suppression when dsRNA was applied at late day period, being most efficient at night, which revealed a diurnal variation in dsRNA treatment efficacy. Exogenous *NPTII*-dsRNA considerably reduced *NPTII* expression in 4-week-old plants and only limited it in 2- and 6-week-old plants. In addition, a more discernible *NPTII* downregulation was detected under low soil moisture conditions. Treatment of adaxial and abaxial leaf surfaces by brushes, spraying, and pipetting showed a higher *NPTII* suppression, while infiltration and inoculation were less efficient. Thus, appropriate plant age, late time of day, low soil moisture, and optimal dsRNA application modes are important for exogenously induced gene silencing.

## 1. Introduction

RNA interference (RNAi) is a natural gene regulation and antiviral defense mechanism that has been actively explored and exploited for both plant gene functional studies and biotechnological applications in disease control and crop improvement [1,2]. During RNAi, long double-stranded RNAs (dsRNAs) are recognized and converted into small fragments of 20–24-nucleotide (nt)-long RNA duplexes, i.e., small-interfering RNAs (siRNAs) or miRNAs, by a ribonuclease DICER [3,4]. These siRNAs are then incorporated into the RNA-induced silencing complex (RISC) to cleave, destabilize, or hinder translation of any homologous mRNAs.

The available RNAi-based technologies of crop improvement and gene functional studies were mainly based on the generation of hairpin RNA (hpRNA)/dsRNA-expressing transgenic plants and host-induced gene silencing [5,6], virus-induced gene silencing (VIGS [7]), insect dsRNA feeding, or foliar dsRNA application to control insect pests [8]. Although there is no conclusive evidence that genetically modified plants could cause adverse health or other effects [9,10], the consequences of genetic modifications of the plant genome are not clear, and this raises serious public concerns. Although VIGS does not require genetic modifications of plants, there are serious limitations that prevent from its wide application in agriculture [11]. Therefore, development of new sustainable and eco-friendly approaches for plant gene regulation without genomic modifications is important.

Exogenous RNAi (exo-RNAi)-based approaches, i.e., via application of dsRNA, hpRNA, or siRNAs onto the plant surfaces, are developed as a promising alternative to transgenic plants and VIGS [6,12,13,14]. There is a relatively high number of studies reporting on plant uptake of externally applied dsRNA, hpRNA, or siRNA targeting specific genes of infecting fungi [15,16,17,18] and viruses [19,20,21,22]. These studies indicated that the exogenous RNAs enter plant vascular system, spread through the plant, enter the infecting fungal pathogen, and trigger host and pathogen RNAi machinery, inducing RNAi-mediated plant resistance. However, there is a limited number of studies reporting on exogenous RNA application for targeting plant endogenes and transgenes (reviewed in [12]). Several studies reported on the plant transgene suppression after exogenous application of dsRNA [20,23], hpRNA [24], or siRNA [24,25,26,27]. According to Numata et al. [25] and Dalakouras et al. [26], transgene silencing after siRNA treatments was successful only after application of accessory technologies (using carrier peptide or high-pressure spraying with a conventional compressor and an air brush pistol). 

To the best of our knowledge, there are only three studies [28,29,30] and one patent [31] reporting on a downregulation of a plant endogenous gene after direct exogenous application of the gene-specific dsRNA molecules without accessory techniques. The patent WO/2011/112570 by Monsanto agricultural company provided the first description of direct application of RNA molecules to plant surfaces to achieve local and systemic target gene silencing. The authors of the patent reported that dsRNAs, siRNAs, ssRNA, and even ssDNA suppressed both transcript and protein levels of 5-enolpyruvylshikimate-3-phosphate synthase (*EPSPS*) gene in tobacco and amaranth leaves. According to Lau et al. [28], mechanical inoculation of crude bacterial extract containing *MYB1*-encoding dsRNAs onto flower buds of the orchid *Dendrobium hybrid* obviously lowered mRNA levels of *DhMyb1* and changed phenotype of floral cells (from conical to flattened epidermal cells). Li et al. [29] targeted several *Arabidopsis* and rice genes, including *Mob1A*, *WRKY23,* and *Actin,* by plant root soaking into dsRNA solution. This approach led to dsRNA absorption and severely reduced mRNA levels of the targeted genes. There were also two studies where a carrier peptide or nanoparticles were used to ensure dsRNA perception and target silencing after external plant treatments. Warnock et al. [30] demonstrated a considerable sequence-specific reduction in mRNA levels of two sugar transporter genes *STP1* and *STP2* in tomato seedlings incubated with the *STP*-dsRNA solution, and this triggered corresponding reductions in sugar levels. According to Jiang et al. [32], application of dsRNA mixed with cationic fluorescent nanoparticles G2 to the root tip of *Arabidopsis thaliana* markedly lowered transcript levels of *SHOOT MERISTEMLESS* (*STM*) and *WEREWOLF* (*WER*) transcription factor genes implicated in shoot apical meristem regulation and root epidermis control. The G2/dsRNA-treated plants exhibited retarded growth and reduced meristem size, while treatment with the only dsRNA did not lead to these effects. There was also a study by Numata et al. [25] in which infiltration of *A. thaliana* leaves with a peptide carrier in a complex with siRNAs targeting the chalcone synthase (*CHS*) gene induced a local loss of anthocyanin pigmentation, but the mRNA or protein levels of CHS were not analyzed.

A relatively high number of studies showed plant perception of exogenous RNA molecules leading to further silencing of the targeted plant pathogen genes, and this is being actively developed and discussed as spray-induced gene silencing (SIGS) technology [33]. However, the exo-RNAi-based silencing was not further actively exploited to regulate plant endogenous genes. It is possible that the selection of appropriate physiological and environmental conditions for plant perception of RNA molecules and optimal dsRNA application modes are critical for exogenously induced RNAi and target gene silencing in plants. In this study, we analyzed the importance of plant age, time of day, environmental stress cues, and application means for dsRNA-induced suppression of the neomycin phosphotransferase II (*NPTII*) transgene, as transgenes are sequences that are more prone to silencing [34,35,36]. The results showed that plant age, late day period, and low soil moisture are the important factors influencing the efficacy of target gene silencing in *A. thaliana*. 

## 2. Results 

### 2.1. The Effect of Plant Age and Time of Day on the dsRNA-Induced NPTII Suppression

To analyze the effect of different physiological conditions on the efficacy of dsRNA-induced transgene silencing, we used two transgenic plant lines of *A. thaliana* (KA0-1 and KA0-2) bearing the *NPTII* transgene under the control of the double CaMV 35S promoter and highly expressing it as established earlier [27]. A large fragment of the *NPTII* gene (599 bp out of 798 bp) was amplified by PCR for further in vitro transcription and dsRNA production. For external application, 35 µg of the synthesized *NPTII*-encoding dsRNA was diluted in 100 µL of water (per individual plant) and directly applied on the leaf surface of 2-, 4-, or 6-week-old *A. thaliana* by spreading with sterile individual brushes (Supplementary video S1). The analysis of the dsRNA concentration effect was performed previously [23], indicating that 35 µg of the dsRNA per plant resulted in the highest *NPTII* silencing efficiency compared to other concentrations.

**Figure 1:**
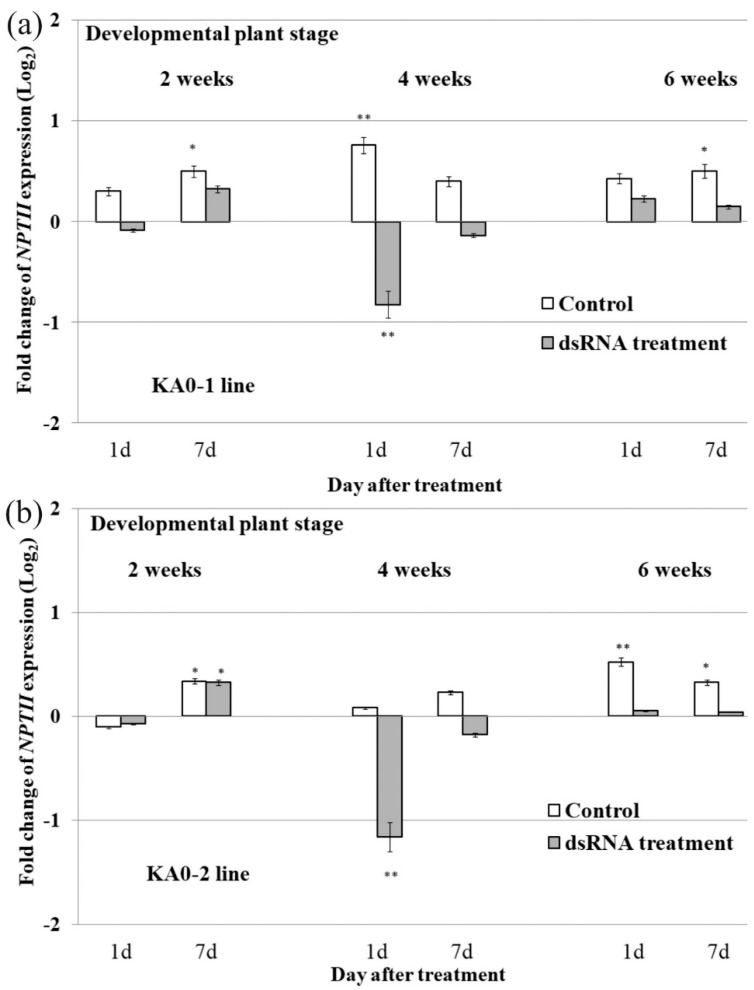
The effect of plant age on the dsRNA-induced *NPTII* downregulation in *Arabidopsis thaliana*. Quantification the *NPTII* mRNAs (log_2_) in the KA0-1 (**a**) and KA0-2 (**b**) lines of *A. thaliana* in response to foliar application of sterile filtered water (control water treatment) or *NPTII*-encoding dsRNA (dsRNA treatment) relative to *NPTII* mRNA level in *A. thaliana* before treatments. The abaxial and adaxial surfaces of the leaves were treated by sterile soft brushes with 0.35 µg/µL of *NPTII*-*ds*RNA or water (100 µL per individual treatment). dsRNA was applied to an individual 4- or 6-week-old plant and to three 2-week-old individuals in an independent experiment. qRT-PCR data are presented as mean ± SE (two independent experiments). *, **—significantly different from the values in plants before treatments at *p* ≤ 0.05 and 0.01, respectively, according to the Student’s *t*-test.

Then, to analyze the effect of plant age on the efficacy of dsRNA-induced *NPTII* suppression, we compared *NPTII* mRNA levels in the leaves of 2-, 4-, and 6-week-old KA0-1 and KA0-2 plant lines before, 1 day and 7 days after the external dsRNA treatment. The data revealed a considerable suppression of NPTII transcript levels in the 4-week-old plants 1 day post-treatment, while no considerable effect on the NPTII mRNA levels was detected after control water treatment (Figure 1 a,b). Foliar dsRNA application to the 2-week-old and 6-week-old A. thaliana plants did not lead to a reduction of NPTII mRNA levels. In this case, both water and dsRNA treatments either increased or did not influence the transgene mRNA levels in the KA0-1 and KA0-2 lines. We also noted that the dsRNA treatment had a deleterious effect on the phenotype and performance of 2-week-old plants (sometimes the plant leaves withered or even died off). Therefore, in further experiments, we used 4-week-old plants.

**Figure 2:**
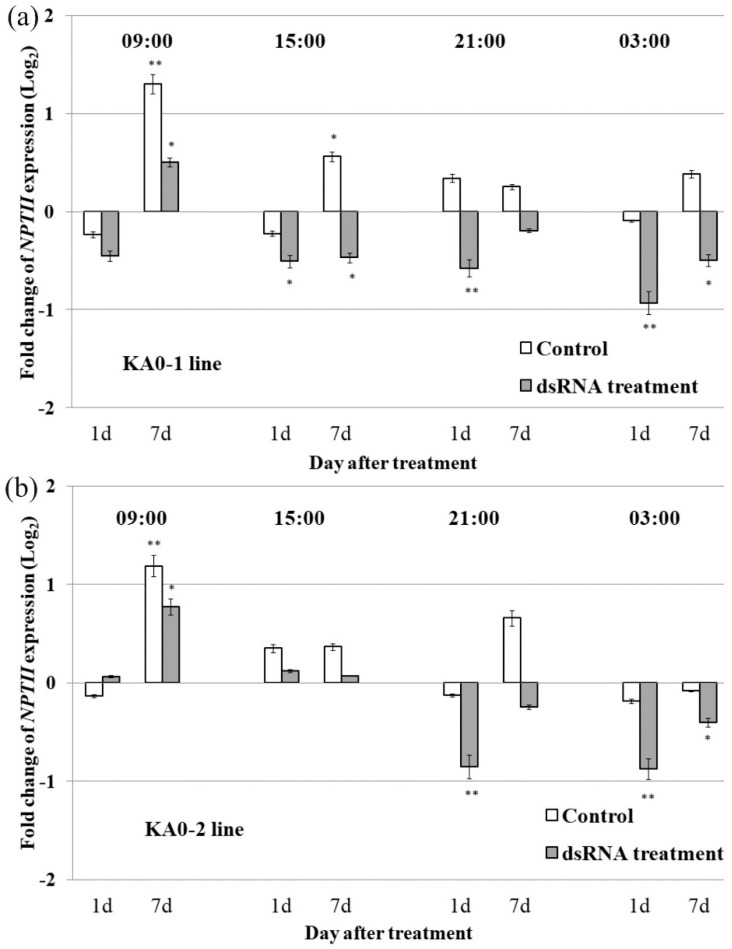
The effect of time of the day on the dsRNA-induced *NPTII* downregulation in 4-week-old *Arabidopsis thaliana*. Quantification the *NPTII* mRNAs (log_2_) in the KA0-1 (**a**) and KA0-2 (**b**) plant lines of *A. thaliana* in response to foliar application of sterile filtered water (control) or *NPTII*-encoding dsRNA (dsRNA treatment) at different times of the day, relative to *NPTII* expression in the same plants before treatments. 09:00, 15:00, 21:00, and 03:00 – time for dsRNA treatment and RNA isolation. The abaxial and adaxial leaf surface of an individual plant was treated by sterile soft brushes with 0.35 µg/µL of *NPTII*-*ds*RNA or water (100 µL per individual plant). RNA was isolated from the same individual plant before, 1 day, and 7 days after treatments (two independent experiments). qRT-PCR data are presented as mean ± SE. *, **—significantly different from the untreated plant at *p* ≤ 0.05 and 0.01, respectively, according to the Student’s *t*-test.

To assess the diurnal variation of the dsRNA treatment efficacy, we analysed *NPTII* transcript abundancy after the dsRNA was applied to 4-week-old *A. thaliana* every 6 h of the day, at 9:00, 15:00, 21:00, and 3:00 (Figure 2a,b). RNA was isolated at 9:00, 15:00, 21:00, and 3:00 before, 1 day and 7 days after the treatments, respectively. Surprisingly, we did not observe an inhibition of NPTII mRNA levels after dsRNA application in the morning at 9:00 for both the KA0-1 and KA0-2 lines. At 9:00, the NPTII transcript abundance remained unaffected (1 d post-treatment) or markedly increased (7 days post-treatment) in both transgenic lines. At 15:00, NPTII transcript levels showed a considerable downregulation only in the KA0-1 line (Figure 2a) and remained essentially unaffected in the KA0-2 line at the same time (Figure 2b). Then, we observed a considerable downregulation of NPTII mRNA levels both in the KA0-1 and KA0-2 lines at 21:00 (1 day post-treatment) and at 3:00 (1 and 7 days post-treatment). Due to the suppression efficacy and time convenience, we have chosen treatment of 4-week-old A. thaliana plants at 21:00 for all of the following experiments.

### 2.2. The Effect of Different dsRNA Application Methods on the NPTII Suppression Efficacy

**Figure 3:**
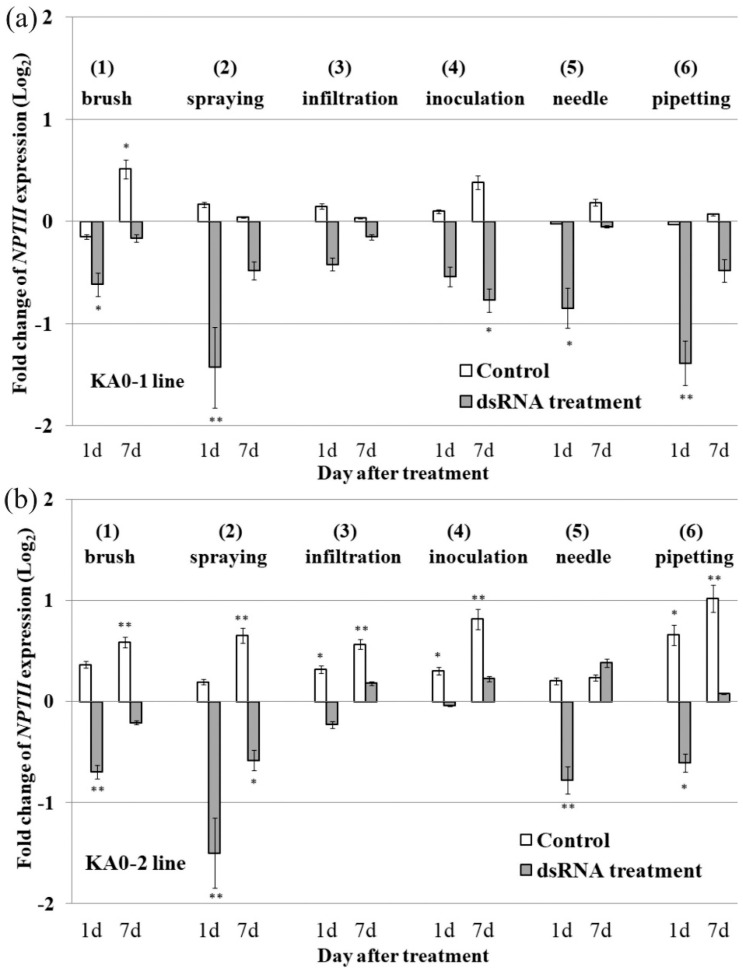
The effect of different dsRNA application methods on dsRNA-induced *NPTII* downregulation in 4-week-old *Arabidopsis thaliana*. Quantification the *NPTII* mRNA levels (log_2_) in the KA0-1 (**a**) and KA0-2 (**b**) lines of *A. thaliana* in response to foliar application of sterile filtered water (control) or *NPTII*-encoding dsRNA (dsRNA treatment) relative to *NPTII* mRNA level in the same plants before treatments. The dsRNAs were applied by (1) spreading with individual soft brushes; (2) spraying with a 2 mL atomizer; (3) syringe infiltration; (4) mechanical inoculation; (5) needle injection; (6) pipetting by an automatic sampler. All leaves of one rosette for each type of condition were treated on both the adaxial (upper) and abaxial (lower) and sides, except for and needle (upper) and infiltration (lower). The leaf surface of each individual plant was treated with 0.35 µg/µL of *NPTII*-*ds*RNA or water (100 µL per individual plant). RNA was isolated from the same individual plant before, 1 day, and 7 days after treatments (two independent experiments). qRT-PCR data are presented as mean ± SE. *, **—significantly different from the untreated plant at *p* ≤ 0.05 and 0.01, respectively, according to the Student’s *t*-test.

To assess and compare the silencing efficiency of different dsRNA application approaches, the *NPTII*-dsRNAs were applied on the foliar surface of 4-week-old *A. thaliana* rosettes by the following methods: (1) spreading with individual soft brushes, (2) spraying with a 2 mL atomizer, (3) syringe infiltration, (4) mechanical inoculation, (5) needle injection, and (6) pipetting by an automatic sampler (Supplementary Videos S1–S6). Brush spreading and pipetting of the *NPTII*-dsRNA at both the adaxial and abaxial leaf surfaces considerably downregulated (1 d after treatment) or limited growth (7 days after treatment) of the *NPTII* both in the KA0-1 and KA0-2 lines (Figure 3a,b). Spraying resulted in a more pronounced inhibition of *NPTII* expression with a downregulation effect both 1 day and 7 day after treatment for the two plant lines. Needle injection of the dsRNA solution into the adaxial (upper) leaf side markedly reduced *NPTII* mRNA levels 1 day post-treatment but did not show a clear and consecutive downregulation or a limiting effect 7 days post-treatment. Infiltration and inoculation either did not considerably affect or only limited growth of the *NPTII* mRNA levels in most cases, except for a marked *NPTII* dowregulation effect 7 days after treatment in the KA0-1 line.

### 2.3. The Effect of Salt, Cold, Heat, and Soil Desiccation on the dsRNA-Induced NPTII Suppression

**Figure 4:**
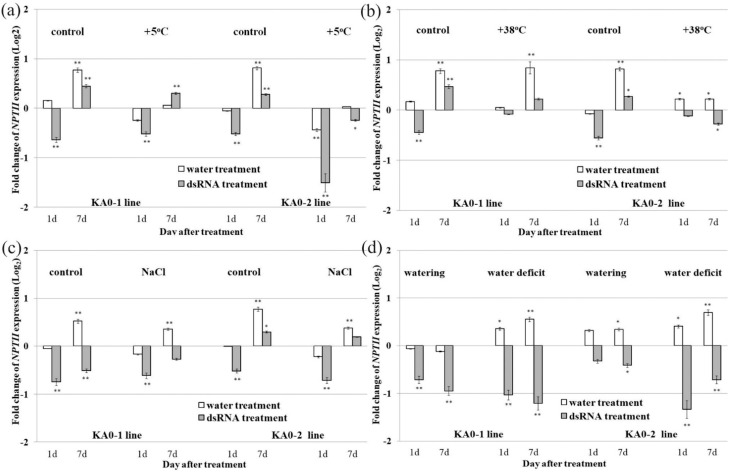
The effect of salt, heat, cold, and soil drying on the dsRNA-induced *NPTII* suppression in 4-week-old lines of *Arabidopsis thaliana*. Quantification the *NPTII* mRNA levels (log2) in the KA0-1 (**a**) and KA0-2 (**b**) lines of *A. thaliana* in response to foliar application of sterile filtered water (water treatment) or *NPTII*-encoding dsRNA (dsRNA treatment) relative to *NPTII* mRNA level in the same plants before treatments cultivated under different stress conditions. (**a**) For cold stress, the plants were stressed at +5 °C for 2 h after the dsRNA treatment; (**b**) for heat stress, the plants were stressed at +38 °C for 2 h after the dsRNA treatment; (**c**) for salt stress, the plants were well-irrigated with 200 mM NaCl solution for 2 h after dsRNA treatment; (**d**) for assaying the importance of soil moisture, the plants were not watered for 3 weeks or well-watered 1 d before the dsRNA treatments. For control conditions (control), the plants were normally cultivated at 22 °C. The abaxial and adaxial surface of the leaves was treated by sterile soft brushes with 0.35 µg/µL of *NPTII*-dsRNA or water (100 µL per individual plant). RNA was isolated from the same individual plant before, 1 day, and 7 days after treatments (two independent experiments). qRT-PCR data are presented as mean ± SE. *, **—significantly different from the untreated plant at *p* ≤ 0.05 and 0.01, respectively, according to the Student’s *t*-test.

We analyzed the *NPTII* expression levels in *A. thaliana* exposed to the most common environmental stress cues, including cold and heat stresses, high salinity, and water deficit (Figure 4a–d). Cold stress applied for 2 h after the foliar application of dsRNA did not exert a consistent and considerable effect on the *NPTII* mRNA levels in the two transgenic plant lines (Figure 4a). While the post-treatment cold stress slightly lowered the dsRNA-induced gene silencing efficacy in the KA0-1 line, it improved the *NPTII* downregulation in the KA0-2 line. The cold stress stimulating effect was detected only 1 day post-treatment. After 7 days, the plants increased the *NPTII* expression, and the stimulating effect have vanished.

High-temperature stress applied for 2 h after the *NPTII*-dsRNA exerted a negative effect on the efficiency of the *NPTII* suppression (Figure 4b). While there was a substantial *NPTII* downregulation effect under control conditions at 1 day time point, we did not observe a downregulation of *NPTII* in the heat-stressed plants, except for that in KA0-2 7 days post-treatment. Thus, it appears that heat stress postponed the *NPTII* suppression effect 1 d post-treatment. Salt stress imposed after application of dsRNA did not influence the efficacy of the dsRNA-induced *NPTII* suppression 1 d post-treatment (Figure 4c). The data show that the alterations were not statistically significant after 7 days for salt-stressed plants. Therefore, salt stress had a slight negative effect on dsRNA treatment efficacy 7 days post-treatment. Then, we compared the effect of the *NPTII-*dsRNA treatments on the *NPTII* transcript levels in plants under high soil moisture (watering 1 day before treatment) and low soil moisture (water withdrawn for 3 weeks) conditions (Figure 4d). The data revealed that plant dsRNA application shortly after watering conditions negatively influenced the efficiency of the dsRNA-induced *NPTII* silencing, although the difference between plants grown in the wet and dry soil was more evident for only KA0-2 transgenic line.

## 3. Discussion

The discovery of the RNAi phenomenon has led to the development of powerful genetic engineering tools for crop improvement, disease management, and plant gene functional studies. Currently, the main RNAi-based approach relies on constructing transgenic plants expressing dsRNAs or hpRNAs designed to silence specific plant or plant pathogen genes for regulating plant properties [5,6,14]. The usefulness of the RNAi-based transgenic technologies in crop improvement allowed researchers to develop seedless fruits, increase fruit shelf life, inhibit the accumulation of plant allergens, or improve plant stress tolerance [5]. However, the consequences of the RNAi technologies relying on stable genetic transformation have raised public discussions and scientific uncertainty on the consequences of permanent genetic modifications. Thus, development of new non-invasive technologies based on exogenous RNA treatments with a gene-specific silencing effect that would not permanently modify a plant genome is an important challenge for biotechnology.

At present, there is a limited number of reports on the direct dsRNA application to the plant exterior surface leading to silencing of specific plant target genes [12,13,14]. In this study, we analyzed the influence of different methods and treatment conditions on the efficacy of transgene silencing in *A. thaliana* after foliar dsRNA application. The available literature presents compelling evidence taken to indicate that plant transgenes are more sensitive to RNAi-mediated silencing than endogenes due to the absence of introns and 5′/3′-untranslated regions and to a higher level of aberrant mRNAs produced and transcribed into secondary dsRNAs [34,35,36]. The results presented in this study showed that appropriate plant age, late time of day, and low soil moisture are important factors ensuring a high efficiency of exogenously induced RNAi in *A. thaliana.* Foliar application of the *NPTII*-dsRNA aqueous solutions reduced the *NPTII* expression at a considerable level in 4-week-old plants, while it was only limited in the 2- and 6-week-old plants. The low effectiveness of the external dsRNA treatments when using the 2-week-old plants was likely caused by the dsRNA deleterious effect on the plant performance, which could result from smaller plant surface area and more delicate plant tissues. In contrast, 6-week-old plants possess larger surface area, and senescence processes were activated, which could render the 6-week-old plant tissues less susceptible to the dsRNA uptake and perception. The present findings also demonstrated a higher *NPTII* suppression at late day period, being most efficient at night, which indicated possible importance of diurnal variation in plant transpiration and lower photosynthetic rates for active dsRNA perception and gene silencing. In addition, a more discernible downregulation effect was detected under low soil moisture conditions, which suggests that the dsRNA was absorbed more efficiently to intensify water supply. It should be noted that the data obtained often show that *NPTII* gene expression increased after the control water treatments. We propose that *NPTII* mRNAs accumulated with time due to either impaired RNA degradation or elevated *NPTII* transcription. In addition, variations in the CaMV 35s promoter activity could contribute to this process. A number of studies revealed that the CaMV 35s promoter is not completely conservative [37,38,39,40]. Expression of CaMV 35S-derived transgenes can markedly vary depending on plant developmental stage, time of day, plant tissue type, and environmental conditions. Our recent results (submitted as a separate study) revealed that the 35s-driven *NPTII* expression gradually increased with the development of the *Arabidopsis* lines.

Taken together, our results demonstrated that the efficacy of exogenously induced transgene silencing varied depending on the plant age, time of day, application means, and soil moisture. Proper selection of the optimal physiological and environmental conditions is important to elicit efficient gene silencing in plants after foliar dsRNA application. According to Goodfellow et al. [41], plant-associated endophytic bacteria have significant potential in respect of further development of exo-RNAi-based plant modifications. Thus, the development of the tools of exogenously induced RNAi could contribute to further achievements in crop improvement and to the implementation of new instruments in plant gene functional studies.

## 4. Materials and Methods

### 4.1. Plant Material

The *NPTII*-transgenic plants of *A. thaliana* ecotype Columbia L. were obtained by floral-dip and characterized previously [27]. Briefly, the plants were transformed with the plasmid construction pZP-RCS2-*NPTII* [42] that carried the *NPTII* transgene under the control of the double 35S promoter of the cauliflower mosaic virus (CaMV 35S). The independent transgenic lines KA0-1 and KA0-2 used in the present study were T_4_ homozygous plants with single-copy *NPTII* insertion.

### 4.2. Plant Growth Conditions

The seeds of wild-type *A. thaliana* were vapor-phase sterilized as described [27] and plated on solid ½ Murashige and Skoog (MS) medium [43] for 2 days at 4 °C. Then, the plates were kept at 22 °C for 1 week in a growth chamber (Sanyo MLR-352, Panasonic, Osaka, Japan) at a light intensity of ~120 μmol m^−2^ s^−1^ over a 16 h daily light period. Then, 1-week-old *A. thaliana* seedlings were planted to pots (7 cm × 7 cm) containing 100 g of commercially available rich soil and were grown under the conditions described under plastic wrap for additional 3 weeks without additional irrigation.

### 4.3. dsRNA Synthesis

A large fragment of the *NPTII* (GenBank AJ414108, 599 bp out of 798 bp) gene was amplified by PCR for in vitro transcription and dsRNA production. PCRs were performed in a T100^TM^ Thermal Cycler (Bio-Rad Laboratories, Inc., Hercules, CA, USA) programmed for an initial denaturation step of 2 min at 95 °C followed by 5 cycles of 10 s at 95 °C, 10 s at 65 °C, 38 s at 72 °C, next step by 35 cycles of 10 s at 95 °C, 48 s at 72 °C, and last step 2 min at 72 °C. Amplification reactions were performed in volumes of 30 µL containing 6 µL of 5× Taq Red Buffer (Evrogen, Moscow, Russia), 0.2 mM of each dNTP, 0.2 µM of each oligonucleotide primer and 2 units of Taq DNA polymerase (Evrogen). dsRNA of *NPTII* was synthesized using T7 RiboMAX™ Express RNAi System (Promega, Madison, WI, USA). The T7 promoter sequence was introduced into both the 5′ and 3′ ends of *NPTII* in a single PCR. The primers are listed in Table S1. The obtained PCR products were used as templates for in vitro transcription and *NPTII*-dsRNA synthesis following the manufacturer’s protocol. The resultant dsRNA was analyzed by gel electrophoresis to estimate its purity and integrity and was spectrophotometrically analyzed to estimate its amount.

### 4.4. dsRNA Application Methods and Conditions

The dsRNAs were applied on the leaf surface of individual *Arabidopsis* rosettes by different application means, including brush spreading, spraying, infiltration, inoculation, needle injection, and pipetting (Supplementary Videos S1–S6). All leaves of one rosette for each type of condition were treated on both the adaxial (upper) and abaxial (lower) sides, except for needle injection (upper side) and infiltration (lower side). The dsRNAs were applied by (1) spreading with individual soft brushes (natural pony hair) sterilized by autoclaving; (2) spraying with 2 mL atomizer polypropylene vials; (3) syringe infiltration avoiding the midvein; (4) mechanical inoculation by gentle rubbing the carborundum-containing inoculation mixture onto the leaves; (5) gentle needle injection; (6) pipetting by an automatic sampler. For all application modes, 35 µg of the dsRNA were diluted in 100 µL of water, except for spraying where 35 µg of dsRNA were diluted in 200 µL of water. To assess the diurnal variation in the dsRNA treatment efficacy, we analysed *NPTII* expression after the dsRNA was applied every 6 h of the day, at 9:00, 15:00, 21:00, and 03:00. dsRNA treatments in all other experiments were carried out at 21:00-21:30. Soil water content before dsRNA treatment was 65 ± 5%, except for waterlogging and water deficit experiments (see below). To assess the effect of plant age on the dsRNA application efficacy, the 2-, 4-, and 6-week-old *A. thaliana* plants were used for dsRNA treatments. For all other experiments, dsRNA was applied to 4-week-old plants.

One plant of the KA0-1 and KA0-2 lines was treated with the *NPTII*-dsRNA (100 µL) or filtered sterile water (100 µL) per each experimental condition in each independent experiment. However, it should be noted that 35 µg of dsRNA caused a negative effect to the physiological state of 2-week-old plants when applied to a single individual (due to plant size limitations and more vulnerable tissues). Therefore, three individual 2-week-old plants were used for RNA isolation at each time point (3 plants were treated with 35 µg of dsRNA or water).

### 4.5. Abiotic Stress Treatments of Transgenic *Arabidopsis*

The plants were subjected to salt, cold, and heat stress treatments as described [44] after the dsRNA treatment for 2 h. For control conditions, the 4-week-old plants were cultivated at 22 °C in a growth chamber as described above. We decided to apply the stress factors for no longer than 2 h in order to avoid premature dsRNA degradation. Briefly, for salt stress, the 4-week-old plants were well-irrigated with 200 mM NaCl solution applied at the bottom of the pots for 2 h after dsRNA treatment. For cold stress, the 4-week-old *A. thaliana* plants were stressed at +5 °C for 2 h after dsRNA treatment in an environmental chamber (KS-200 SPU, Smolensk, Russia). For heat stress, the 4-week-old plants were stressed at +38 °C for 2 h after dsRNA treatments in the environmental chamber. After 2 h of stress treatments, the plants were normally cultured at 22 °C before RNA isolation. For assaying the importance of soil moisture condition, the plants were either cultivated without additional irrigation and plastic wrap (water deficit, soil water content 60 ± 5) or were well-watered 1 day before the dsRNA treatments (watering, soil water content 80 ± 5%). We watered plants 1 day in advance to allow the excessive water leak out and to wait until plants adapt to the well-watered conditions.

### 4.6. RNA Isolation and Reverse Transcription

For nucleic acid isolation, a typical adult leaf of 4- or 6-week-old *A. thaliana* was collected from the same individual plant at all time points (before, 1 day and 7 days post-treatments) for each type of treatment in an independent experiment. For 2-week-old plants, RNA was isolated from three individuals for each time point (before, 1 day and 7 days post-treatment) due to plant size and vulnerability limitations. To assess the diurnal variation in the dsRNA treatment efficacy, RNA was isolated at 9:00, 15:00, 21:00, and 3:00 before, 1 day and 7 days after the treatments, respectively. For all other experiments, RNA isolations were carried out at 21:00.

The isolation of total RNA was performed using the cetyltrimethylammonium bromide (CTAB)-based protocol [45] and complementary DNAs were synthesized as described [46].

### 4.7. Gene Expression Analysis by qRT-PCR

The reverse transcription products were amplified by PCR and verified for the absence of DNA contamination using primers listed in Table S1. The qRT-PCRs were performed with EvaGreen Real-time PCR (Biotium, Hayward, CA, USA) as described in [47,48] using two internal controls (GAPDH and UBQ) selected in previous studies as relevant reference genes for qRT-PCRs on *Arabidopsis* [49]. The expression was calculated by the 2^−ΔΔCT^ method [50]. All GenBank accession numbers and primers are listed in Table S1.

### 4.8. Statistical Analysis

The data are presented as mean ± standard error (SE) and were tested by paired Student’s *t*-test. The *p* < 0.05 level was selected as the point of minimal statistical significance in all analyses. Two independent experiments were performed for each type of experiment.

## Data Availability

The data presented in this study are available within the article and supplementary material.

## Supplementary Materials

Supplementary materials can be found at https://www.mdpi.com/2223-7747/10/2/264/s1. Table S1 Primers used in PCR and qRT-PCR amplification, Video S1: Brush, Video S2: Spraying, Video S3: Infiltration, Video S4: Inoculation, Video S5: Needle, Video S6: Pipetting.
