# Supplementary material for: Physiological Conditions and dsRNA Application Approaches for Exogenously induced RNA Interference in Arabidopsis thaliana
Source: Plants (Basel). 2021 Jan 30;10(2):264. doi: 10.3390/plants10020264 (PMC7911504; doi:10.3390/plants10020264)
Supplement: Supplementary file 1 [file plants-10-00264-s001.zip › Supplementary-2/Table S1.docx]

**Table S1** Primers used in RT-PCR and qRT-PCRs.

| Gene name  (GenBank acc. no) | Primer name | Primers, 5′-3′ |
| --- | --- | --- |
| Primers for cDNA check-up on DNA contamination, 5′-3′ | | |
| AtGAPDH  (NM_111283) | AtGapdh-s  AtGapdh-a | 5′CTG GAA TGT CTT TCC GTG TC  5′ATT CGT TGT CGT ACC ATG AC |
| Primers for PCR and real-time PCR, 5′-3′ | | |
| *NPTII* (AJ414108) | nptII-realS  nptII-realA | 5′ttgctgaagagcttggcggcgaat  5′TCAGAAGAACTCGTCAAGAAGG |
| AtGAPDH  (NM_111283) | AtGapdh-real-s  AtGapdh-real-a | 5′TTG GTG ACA ACA GGT CAA GCA  5′AAA CTT GTC GCT CAA TGC AAT |
| AtUBQ  (NM_001084884) | AtUBQ-realS  AtUBQ-realA | 5′GGCCTTGTATAATCCCTGATGAATAAG  5′AAAGAGATAACAGGAACGGAAACATAGT |
| Specific primers for dsRNA design, 5′-3′ | | |
| *NPTII* (AY818371) | npt-T71-s  npt-T72-a | 5′TAATACGACTCACTATAGGGAGAATGTGGATTGAACAAGATGGATTG  5′TAATACGACTCACTATAGGGAGATCCACCATGATATTCGGCAAGCAG |
